# In Vivo Methods to Monitor Cardiomyocyte Proliferation

**DOI:** 10.3390/jcdd9030073

**Published:** 2022-03-03

**Authors:** Alexander Young, Leigh A. Bradley, Matthew J. Wolf

**Affiliations:** Robert M. Berne Cardiovascular Research Center, Department of Medicine, University of Virginia, Charlottesville, 415 Lane Road, Medical Research Building 5, Charlottesville, VA 22908, USA; apy5h@virginia.edu (A.Y.); lab8f@virginia.edu (L.A.B.)

**Keywords:** cardiomyocyte cycling, mouse, transgenics, heart, regeneration

## Abstract

Adult mammalian cardiomyocytes demonstrate scarce cycling and even lower proliferation rates in response to injury. Signals that enhance cardiomyocyte proliferation after injury will be groundbreaking, address unmet clinical needs, and represent new strategies to treat cardiovascular diseases. In vivo methods to monitor cardiomyocyte proliferation are critical to addressing this challenge. Fortunately, advances in transgenic approaches provide sophisticated techniques to quantify cardiomyocyte cycling and proliferation.

## 1. Introduction

Adult mammalian cardiomyocytes, the muscle cells of the heart, have limited ability to re-enter the cell cycle and even lower rates of division to produce new cardiomyocytes. The inability of adult cardiomyocytes to cycle and proliferate significantly impacts myocardial responses to injuries. For example, cardiomyocytes are lost during myocardial infarctions even with successful reperfusion, and this loss contributes to adverse left ventricular remodeling, ischemic cardiomyopathy, heart failure, arrhythmia, and death. Heart failure affects 5.7 million Americans with a projected 46% increase in prevalence by 2030, has a ~50% mortality rate at five years despite current medication and device-based therapies, and accounts for ~2 million physician office visits and ~USD 30 billion in direct medical costs annually [1]. FDA-approved medications to treat heart failure fall within a few classes designed to dampen the adrenergic (e.g., beta-adrenergic receptor antagonists) or renin-angiotensin signaling systems (e.g., ACE inhibitors, ARBs) [2]. Unfortunately, current therapies can only slow or reverse isolated aspects of heart failure. Moreover, there are no reliable therapies available to replace the cardiomyocytes lost to myocardial infarction.

Identifying the drugs that enhance cardiomyocyte proliferation after injury will be groundbreaking, address unmet clinical needs, and represent new strategies to treat cardiovascular diseases. However, methods are needed to quantify adult cardiomyocyte cycling and replication accurately [3]. Rigorous practices are fundamental to investigate the mechanisms that prevent cycling events under conditions of normal growth, aging, and response to injury, and establish new therapies that may enhance cardiomyocyte cycling to improve myocardial function. We will review the in vivo methods to monitor cardiomyocyte proliferation, focusing on transgenic mice.

### 1.1. Normal Mammalian Heart Cycling, Exit from the Cell Cycle, and Estimates of Cardiomyocyte Turnover in Human Hearts

Mammalian cardiomyocytes exit the cell cycle during postnatal heart growth. In mice, the majority of cardiomyocytes are determined within the first week of postnatal life, followed by two waves of DNA synthesis leading to binucleation and increased ploidy, a measure of genome size [4]. A small fraction of cardiomyocytes re-enter the cell cycle in response to injury [5,6,7,8,9,10,11,12,13]; however, cytokinesis is incomplete after cardiomyocytes re-enter the cell cycle, leading to further polyploidization and limited generation of new cardiomyocytes [14]. Human cardiomyocyte turnover is ~0.04% in the first year of life and ~0.01% per year in adulthood based on nuclear bomb test-derived radioisotope decay estimates [4,5,15,16]. Cardiomyocyte cycling increases to ~0.1% after an injury; however, the turnover is likely overestimated because of polyploidy [14,17].

The detection of endogenous expression of cell cycle proteins (Ki67 [18,19,20], Aurora kinase B [21,22,23], Histone H3 phosphorylation [24,25], Anillin [26,27,28], among others), when combined with nucleotide analog incorporation into genomic DNA during S-phase, has been used to estimate cardiomyocyte cycling [3,14]. Measurements of cycling cardiomyocytes based on marker expression range from 0% to 0.8% and 0.01% to 3.8% for uninjured and injured myocardium, respectively [7,29,30]. Measurements of nucleotide analog incorporation using Bromodeoxyuridine, 5-Ethynyl-2-deoxyuridine, or 13N-thymidine into mammalian cardiomyocytes range from 0% to 0.6% in uninjured models and 0.015% to 3.2% after injury [4,10,12,13,29,30,31,32]. Derks and Bergman recently produced a table describing the fractions of labeled cardiomyocytes in different models [14]. The low rates of cardiomyocyte cycling present challenges to their detection and manipulation by transgenic methods. This is particularly important because Bromodeoxyuridine-labeled cardiomyocytes are vastly outnumbered by Bromodeoxyuridine-labeled non-myocytes (fibroblasts, inflammatory cells, etc.). However, quantification of nucleotide analog incorporation into cycling cardiomyocytes can be achieved using cardiomyocyte markers and careful approaches, as described by the Field group [12,33,34,35].

The incorporation of thymidine analogs and transgenic mice revealed spatial and regional distribution of cycled cardiomyocytes after injuries, such as myocardial infarction [6,7,10,34]. Generally, most cardiomyocyte cycling occurs in the border zones, defined as the regions of the heart nearest the infarcted area. The remote zone, defined as the region most distant form the infarct, usually has few cycling cardiomyocytes. The infarcted region may have cycling cardiomyocytes depending on the type of myocardial infarction. Permanent left anterior descending artery occlusion creates a full thickness, thin scar of collagen, myofibroblasts, and few surviving cardiomyocytes. In contrast, the limited occlusion of the left anterior descending artery followed by reperfusion produces a limited infarcted area that is not full thickness and has many surviving cardiomyocytes. Understanding the type of myocardial infarction is necessary to interpret the literature pertaining to cardiomyocyte cycling and cardiac regeneration.

Additionally, the accurate measurement of proliferating cardiomyocytes requires detecting cytokinesis and daughter cells derived from replication, an additional challenge because cycling cardiomyocytes also undergo endoreplication and multinucleation. Many of the transgenic methods described below are based on cell cycle protein expression and can effectively label cycling events, and sometimes karyokinesis, the process of nuclei division to produce bi- and multinucleated cardiomyocytes. However, few reporters have labelled cytokinesis. Therefore, methods that label the clonal expansions of daughter cells are often used to infer cardiomyocyte proliferation.

### 1.2. Cardiomyocyte Endoreplication and Binucleation vs. Proliferation

**Figure 1:**
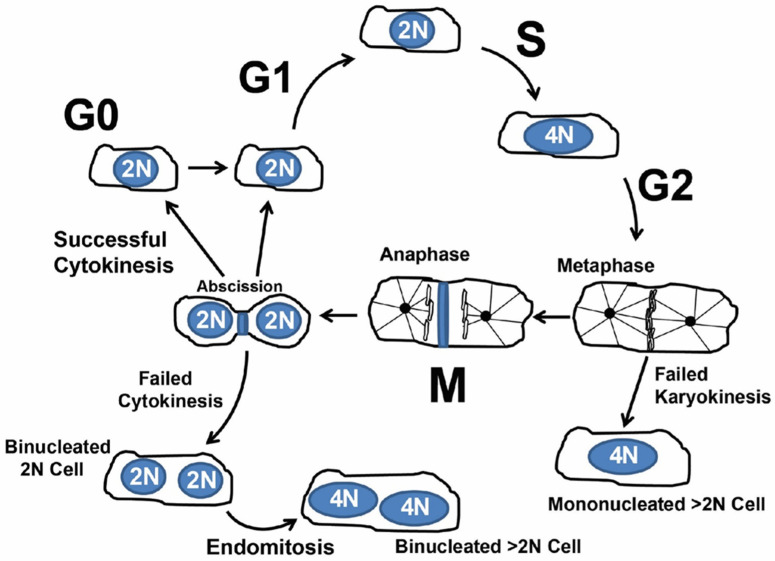
Simplified overview of cardiomyocyte cycling. After G2, failed karyokinesis (nuclear division) produces a single nucleus with >2N genomic DNA content. After mitosis and karyokinesis failed cytokinesis produces a binucleated cell. Further endomitosis of binucleated (on multinucleated) cells produces nuclei with >2N genomic content.

Under normal growth conditions and in response to stress and injury, mammalian cardiomyocytes can reenter the cell cycle and undergo increased ploidy, a measure of genomic DNA content (Figure 1) [14]. The increase in ploidy, also called polyploidization, occurs through endoreplication (also called endoreduplication), in which a cell proceeds through the four canonical phases of the cell cycle. However, cytokinesis is incomplete, producing bi-nucleated or mono-nucleated polyploid cells. Additionally, incomplete cytokinesis and karyokinesis produce nuclei that have greater than 2N ploidy. Deciphering the signal pathways and proteins that a cell uses to drive proliferation and endoreplication are being actively investigated.

Polyploidy occurs throughout the plant and animal kingdoms [36,37]. Teleologically, polyploidization serves two possible objectives in nature. First, an increase in gene copy number provides increased protein products needed for the specialized cell function. Second, an increase in gene copy number may protect a cell from environmental stresses (i.e., radiation exposure) that could inactivate genes essential to cell survival and lead to detrimental effects in an organism [38,39,40,41].

Cardiomyocyte polyploidization can occur in hypertension, cardiac hypertrophy, and myocardial infarction [5,42,43,44]. Cardiomyocyte binucleation may be a barrier to proliferation, limiting mammalian myocardial regeneration. Recently, we observed that ~90% of cardiomyocytes reentering the cell cycle did not produce neighboring cells, suggesting that cycling failed to proliferate and may have undergone endoreplication [6]. These observations were consistent with prior results in the field. Whether the endoreplicative events resulted in complete genome duplications is unclear. Certainly, investigating the mechanisms that control polyploidy will lead to new insights into targets to promote controlled cardiomyocyte proliferation as a therapy to treat cardiovascular diseases, but in vivo methods are necessary to quantify cycling events accurately.

### 1.3. Concept of Actively Cycling and Previously Cycled Cardiomyocytes and Overview of “Snapshot” vs. “Integration of Events” Using an Indelible Mark

**Figure 2:**
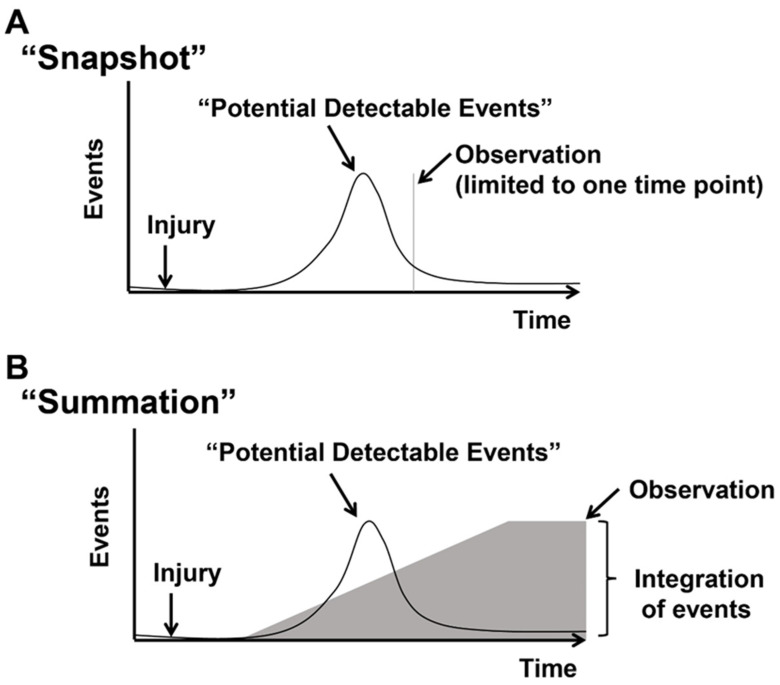
Detecting “Actively cycling” and “Previously cycled” cells. Events represent cycling cells. After an injury, there is an increase and decrease in cycling cells. (**A**) The “snapshot” approach measures cycling events in blue at a distinct time point after the injury. Typically, cycling cells are detected by the co-localization of markers of cycling (Ki67, PH3, Aurora B, and Anillin) with cardiomyocytes or transgenic mice that report phases of the cell cycle (FUCCI, Ki67-RFP, eGFP-Anillin). (**B**) The “summation” approach relies on indelibly marking cycling events and measuring total events after an injury. Cycled cells are labeled by incorporating nucleotide analogs into synthesizing genomic DNA (3H-Thymidine, Bromodeoxyuridine, or 5-Ethynyl-2-deoxyuridine) or transgenic mice that mark cells that cycled or underwent mitosis (*MADM*, Confetti/Brainbow, and *αDKRC::RLTG*). Figure adapted from Bradley et al., Circ Res 2021, 128, 155–168.

The literature regarding the detection of cycling cardiomyocytes in vivo describes various methods that we will review below. The methods can be broadly divided into those that label “actively cycling” and “previously cycled” cardiomyocytes. (Figure 2A,B). Measuring actively cycling cardiomyocytes provides a snapshot of events at the time of tissue analyses based on the colocalization of markers expressed in cycling cells, including phospho-histone-3, Ki67, or Aurora B. These markers are expressed when a cell is actively cycling. Therefore, the detection is dependent on the time tissue is analyzed after injury and can lead to an over or underestimation of cycling events. Current methods cannot distinguish endoreplication and proliferation, although markers specific to the midbody of cytokinesis may suggest proliferative events.

The second approach relies on indelibly marking cells that have cycled and provides a summation measurement of cycling events. Typically, this approach measures the incorporation of thymidine analogs (Bromodeoxyuridine or 5-Ethynyl-2-deoxyuridine) into DNA during S-phase using antibodies or Click-It chemistry to identify cycling cells [32,45,46]. The dose, route of administration, and duration of exposure of Thymidine analogs vary among published investigations. Pulsed dosing or continuous administration of Bromodeoxyuridine or 5-Ethynyl-2-deoxyuridine must be considered when designing and interpreting cell cycling. The disruption of vascularity that occurs in the setting of injury potentially limits the access of Bromodeoxyuridine or 5-Ethynyl-2-deoxyuridine to regions of the myocardium, confounding the interpretation of cycling cells. Moreover, nucleotide analog incorporation occurs during S-phase and cannot distinguish between endoreplication (polyploidy) and proliferation of cardiomyocytes, although careful measurements of nuclear DNA content and nuclei number such as combined with FACS can provide an overall estimation of endoreplication and proliferation. However, the use of dissociated cells, or isolated nuclei, results in the loss of information regarding the spatial distribution of cells in tissues.

This review will focus on recent advances in transgenic mice designed to monitor in vivo cardiomyocyte cycling and proliferation.

## 2. Transgenics Reporter Mice of Actively Cycling Cells

**Figure 3:**
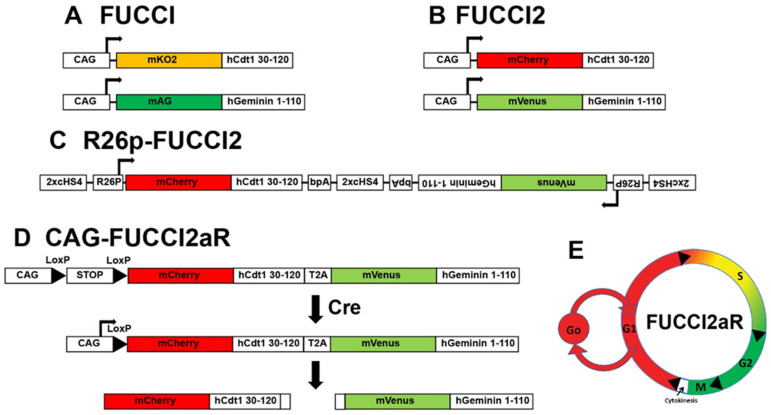
Fluorescent Ubiquitination-based Cell Cycle Indicator (FUCCI) reporters. First-generation FUCCI (**A**) and FUCCI2 (**B**). Second-generation Bidirectional R26p-FUCCI2 (**C**) and polycistronic FUCCI2aR (**D**). (**E**) Schematic of cell cycle phase-dependent differential expression of FUCCIs.

Fluorescent Ubiquitination-based Cell Cycle Indicator (FUCCI) reporters. During regular cell cycling, proteins are targeted for degradation through orchestrated ubiquitination reactions. Two E3 ligases responsible for ubiquitin reactions, APC^Cdh1^ and SCF^Skp2^, have significant roles in targeting proteins for degradation during cell cycle phases [47,48,49,50,51,52]. SCF^Skp2^ is a substrate and a direct inhibitor of APC^Cdh1^, creating reciprocal oscillations such that APC^Cdh1^ is active in late M and G_1_ phases while SCF^Skp2^ is active in S and G_2_. Moreover, the proteins Cdt1 and Geminin are substrates of SCF^Skp2^ and APC^Cdh1^, respectively, and undergo coordinated ubiquitination and degradation, causing Cdt1 and Geminin to be expressed during the G_1_ to S phase and S phase to G_2_/M. Therefore, the creation of fluorescent protein chimeras that harbor Cdt1 or Geminin tags recognized by SCF^Skp2^ and APC^Cdh1^ serve as probes to identify specific cell cycle phases in cultured cells and in vivo. These chimeras are referred to as Fluorescent Ubiquitination-based Cell Cycle Indicator (FUCCI) reporters [49] (Figure 3A).

The first-generation FUCCI probe pairs were separate reporters of fusion of monomeric Kusabira Orange (mKO2) with a truncated human Cdt1 (hCdt1) containing amino acids 30-120 and monomeric Azami Green (AG) and the 110 amino acid N-terminus of the human Geminin (hGem) protein [49]. The transgenes were driven by a constitutively active synthetic CAG promoter containing the cytomegalovirus (CMV) early enhancer element, the promoter and the first exon and the first intron of the chicken beta-actin gene, and the splice acceptor of the rabbit beta-globin gene [53]. The mKO2-hCdt1(30/120) reporter accumulates during the G_1_ phase and is degraded at the G1-S transition. The mAG-hGem(1/110) probe accumulates during S/G_2_/M phases and is degraded prior to cytokinesis. The next generation of FUCCI reporters consisted of mCherry-hCdt1 (30-120) and mVenus-hGem (1-110) [54] (Figure 3B).

The initial FUCCI reporter mice were generated from two lines, CAG-mKO2-hCdt1(30/120) and CAG-mAG-hGem(1/110), each produced by conventional transgenesis [49]. Since the CAG promoter is constitutive, approaches using the alpha-myosin heavy chain (αMHC) promoter to drive FUCCI have been described to provide cardiomyocyte-specific reporter expression [55]. In this case, a mix of αMHC-mKO-hCdt1 and αMHC-AG-hGem1 was co-injected to create transgenic mice. The first-generation CAG- and αMHC-FUCCI mice provided important insights into cycling in vivo; however, there are significant limitations. First, the random insertion of transgenes is associated with positional variegation, causing problems with the amounts of expressed reporters. Positional variegation is a significant issue because the accurate identification of the cell cycle phases depends on each reporter’s expression levels, and baseline differences in expression attributed to the transgene insertion site may confound the results.

The second-generation FUCCIs were designed to avoid the problems of positional variegation associated with separate transgenesis of individual reporters. Bidirectional R26p-Fucci2 mice harbor bidirectionally conjugated mCherry hCdt1(30/120) and mVenus-hGem(1/110), each driven by the constitutive R26 promoter and separated by chicken hypersensitive site 4 (cHS4) transcriptional insulators [54] (Figure 3C). The bidirectional R26p-Fucci2 mice had low expression of the reporters and were lethal as homozygotes.

Newer-generation FUCCIs (FUCCI2a) harbor mCherry hCdt1(30/120) and mVenus-hGem(1/110) as a polycistronic transgene with the probes separated by a *Thosea asigna* virus 2A peptide (T2A) cleavage site [56] (Figure 3D,E). The Fucci2a is driven by a Cre recombinase-inducible CAG promoter and targeted to the mouse Rosa26 locus (designated Fucci2aR mouse). The Fucci2aR mouse provides a robust expression of an inducible FUCCI2a with initial equimolar concentrations of each probe, whose subsequent levels are dictated by the cell cycle phases.

*αMHC-Cre::Fucci2aR*, when used with thymidine analog incorporation into synthesizing DNA, can facilitate the detection of actively cycling and previously cycled cardiomyocytes. In combination with Bromodeoxyuridine incorporation into genomic DNA during the S phase and counterstains for cytokinesis (Aurora B), Fucci has the power to detect one aspect of in vivo proliferation. We generated and characterized cardiomyocyte-specific reporter *αMHC::Fucci2aR* mice and examined Fucci expression in postnatal hearts during the window when cardiomyocytes transition from proliferation to polyploidy [6]. Mono-nucleated cardiomyocytes in G_1_, S, and G_2_/M were seen in early postnatal hearts and transitioned to bi-nucleated G_0_/G_1_ cardiomyocytes by P10. Cardiomyocytes of adult hearts were almost exclusively mCherry+/mVenus- (red) with the rare presence of mVenus+ cells. Our observations of postnatal *αMHC::Fucci2aR* hearts were consistent with postnatal cardiomyocyte growth described in the literature. Although *αMHC::Fucci2aR* mice label actively cycling cells, the approach is limited by the scarcity of actively cycling cardiomyocytes in adult hearts after injury and the inability to label daughter cells to identify proliferative and endoreplicative events.

*Mki67^TagRFP^* mice. The Ki67 protein was initially defined by a monoclonal antibody Ki-67, generated by immunizing mice with nuclei of the Hodgkin lymphoma cell line L428 [18,19,20]. The name was derived from the city of origin (Kiel) and the original clone number from a 96-well plate. Ki67 is a nucleolar protein expressed when cells enter the cycle during G_1_-S-G_2_-M phases but absent in the G_0_. The Ki-67 antigen is present in all proliferating cells, both standard and tumor cells, and therefore a marker of the growth fraction of cell populations. Antibodies against the Ki-67 protein serve as diagnostic tools in different types of neoplasms, immunohistochemically stratify tumors and identify cells committed to entering the cell cycle.

Basak and colleagues created *Mki67^TagRFP^* mice by knocking a TagRFP red fluorescent protein in frame with the C-terminus of the Ki67 coding sequence to label actively cycling cells [57,58]. The percentage of Ki67-RFP+ cells in the hearts of one-week-old *Mki67^TagRFP^* mice was ~10%, and less than 0.05% of Ki67-RFP+ cells were detected from the hearts of adult mice. The *Mki67^TagRFP^* mice were subsequently used to profile proliferative cells in injured mouse hearts. Ki67-RFP+ cells doubled seven days after permanent left anterior descending (LAD) artery ligation myocardial infarction and reached 1% enrichment two weeks after the injury. Of note, TagRFP is expressed in any cell type undergoing cycling, a limitation of *Mki67^TagRFP^* mice to quantify cycling cardiomyocytes. However, careful co-localization of cardiomyocyte-specific markers improves analyses.

**Figure 4:**
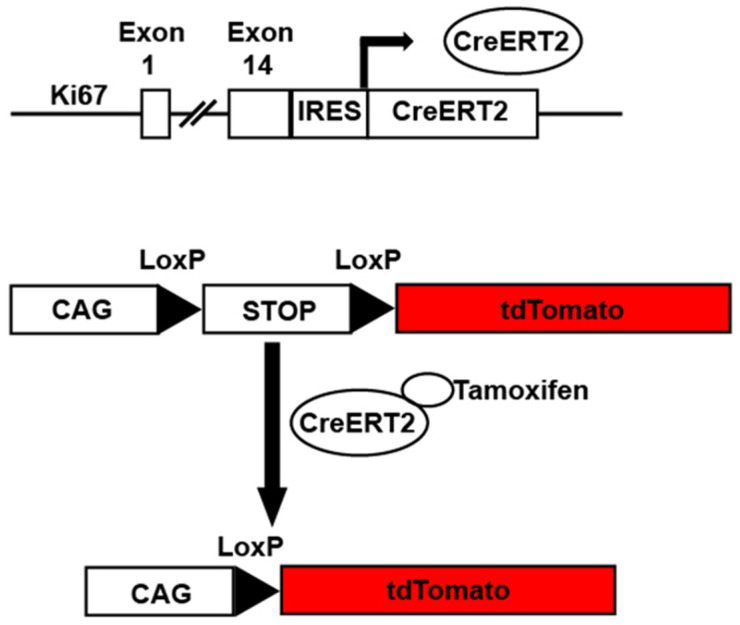
*Ki67^iresCreER^* mice. Example of *Ki67^iresCreER^::CAG-Lox-STOP-Lox-tdTomato* mice. In the presence of tamoxifen, cells undergoing cycling as defined by Ki67 expression have activated Cre recombinase. The activated Cre excises a STOP cassette allowing tdTomato expression under the control of the constitutively active CAG promoter.

*Ki67^iresCreER^* mice. Basek et al. also created *Ki67^iresCreERT2^* mice by knocking in a tamoxifen-inducible Cre recombinase (CreERT2) linked to an internal ribosomal entry sequence (IRES) to drive Cre expression in cycling cells when treated with Tamoxifen [58] (Figure 4). Experiments used *Ki67^iresCreERT2^::Lox-STOP-Lox-tdTomato* mice to label all cycling cells in the heart one day and 1.5 years after Tamoxifen treatment. No tdTomato+ cardiomyocytes were detected one day after treatment. Few tdTomato+ cardiomyocytes (~0.16%) were detected after 1.5 years. However, the majority of proliferating cells in the myocardium were CD31+ endothelial cells, PDGFRα+ fibroblasts, and CD45+ hematopoietic cells, suggesting a continuous cellular turnover of non-cardiomyocyte lineages in the adult homoeostatic murine heart. Thus, the *Ki67^iresCreER^* mice provide another potential strategy to label cycling cardiomyocytes but share limitations similar to the *Mki67^TagRFP^* mice, namely that any cell undergoing cycling will be labeled. Therefore, careful co-localization of the tdTomato signal to cardiomyocytes is needed to avoid the overestimation of cycling events.

**Figure 5:**
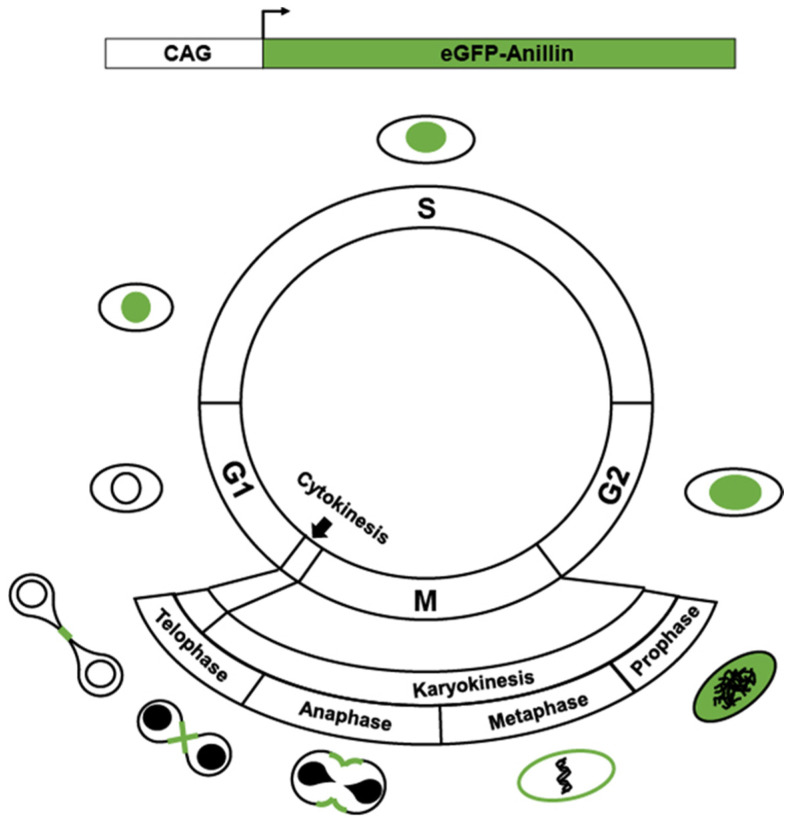
*CAG-eGFP-Anillin*. The subcellular localization of constitutive expression of eGFP-Anillin marks cells that undergo cytokinesis by labeling the mid-body. Figure adapted from Hesse, M. et al., Nat Commun 2012, 3, 1076.

Enhance Green Fluorescent Protein *(eGFP)-Anillin* mice. Anillin is an integral component of the contractile ring during cytokinesis and undergoes cell cycle-dependent changes in a subcellular location [26,27,28]. During the late phase of G_1_-, S- and G_2_-phase of the cell cycle, Anillin is located in the nucleus and then transitions to the cytoplasm and cell cortex in the early M-phase before the contractile ring and mid-body during cytokinesis. After mitosis, Anillin undergoes ubiquitination in early G_1_ by the anaphase-promoting complex associated with Cdh1 and degraded by the proteasome [26,27,28]. Based on the cell cycle-dependent subcellular location, particularly to the contractile ring and midbody during cytokinesis, Anillin serves as a potential substrate for creating transgenics to identify proliferating cells. Hesse and colleagues generated transgenic mice (*CAG-eGFP-Anillin*) that ubiquitously expressed a chimeric of the mouse Anillin cDNA fused to the C-terminus of eGFP under the control of the CAG promoter [59] (Figure 5). The adult *CAG-eGFP-Anillin* mice underwent cryoinjuries of the left ventricular or permanent LAD artery ligation myocardial infarctions, and *eGFP–anillin*-expressing cells were quantified. Four days after the injuries, most *eGFP–anillin*-expressing cells were identified as myofibroblasts by α-smooth muscle actin + staining with ~8% having the *eGFP-Anillin* localized to the contractile ring or midbody. Very few cardiomyocytes in the border zones of the injured myocardium expressed *eGFP-Anillin*, and none of the *eGFP–Anillin*+ signal was located in the midbody or contractile ring. The data suggest that cardiomyocytes in the adult heart did not divide in response to injury. Instead, the cells re-entered the cell cycle without progressing to cytokinesis. Confocal microscopy and quantification of cardiomyocyte DNA content, using endothelial cells as a standard of 2C DNA content, identified that most eGFP–Anillin-negative cardiomyocytes had a 4C DNA content, whereas eGFP–anillin-positive cardiomyocytes had a DNA content between 4C and 8C. The results were consistent with cardiomyocytes undergoing endoreduplication after injury to the myocardium. Similar to the above-described transgenic mice, *eGFP-Anillin* mice label all cycling cells and require careful co-localization of the eGFP-Anillin to cardiomyocytes to avoid the potential overestimation of cycling events.

*Aurora kinase B (Aurkb)-ER ^Cre/+^* mice. Aurkb is a component of the chromosomal passenger complex and localizes to the centromeres and midbody during mitosis and cytokinesis, respectively [21,22,23]. Antibody staining of Aurkb in histological samples is often used to quantify mitotic events. Recently, *Aurkb-ER Cre/+* mice have been generated by targeting a Cre-Ert2 cassette into the start codon of the Aurkb locus to induce the expression of Cre recombinase in cells undergoing mitosis during tamoxifen administration [60]. The advantage of the *Aurkb-ER Cre/+* mice is that tamoxifen activation of Aurkb-ER Cre labeled proliferating cells during development and adult stem and progenitor cells, but not post-mitotic cell in vivo. However, limitations of the *Aurkb-ER Cre/+* mice include the potential need for continuous Tamoxifen administration to ensure induction of the Cre recombinase and the generalized expression of Cre in any cell type that is undergoing mitosis, thus requiring co-localization of cardiomyocytes markers for accurate quantification of events.

## 3. Transgenic Reporter Mice of Previously Cycled Cells

**Figure 6:**
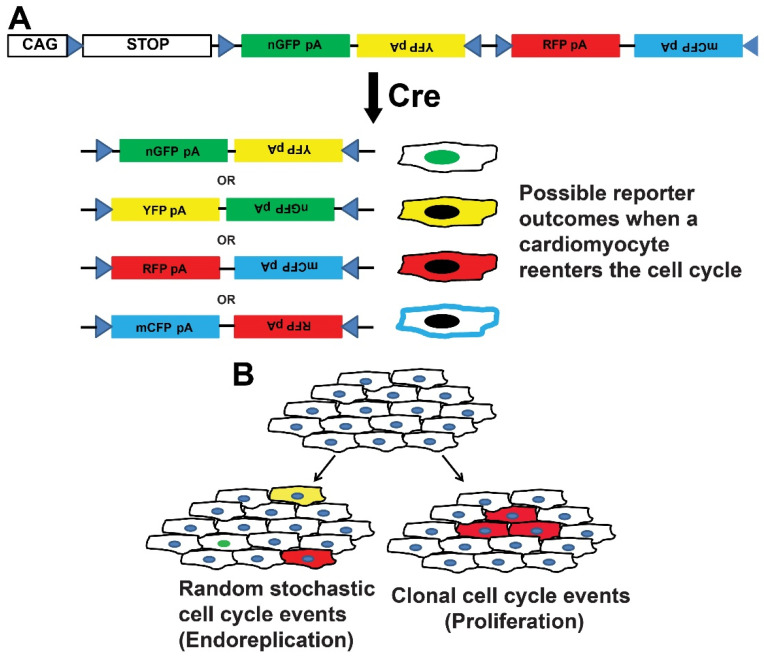
CAG-loxP-STOP-loxP-Confetti (Brainbow). (**A**) The tamoxifen-induced expression of Cre-dependent causes stochastic recombination of a multi-fluorescent reporter. Identifying of clusters of cells expressing the same fluorescent reporters are used to infer clonal expansion. (**B**) Schematic of the interpretation of Confetti expression used to identify random stochastic events (**left**) and clonal expansion consistent with proliferation (**right**).

BrainBow/Confetti mice. Fluorescent protein expression is fundamental to in vivo investigations of cells. Advances in the combinatorial expression of fluorescent reporters using Cre-Lox technology facilitate the detection of clonal expression of proliferating cardiomyocytes. Livet and colleagues first described a genetic strategy, called Brainbow, for stochastic expression of multiple fluorescent proteins from a single transgene [61]. The technology used a series of tandem and inverted LoxP, LoxN, and Lox2272 sites flanking different fluorescent cassettes (cytosolic RFP, nuclear-localized GFP, cytosolic YFP, and membrane-associated CYP) that recombine stochastically in the presence of Cre recombinase (Figure 6).

Using Brainbow, the combinatorial expression of the different pairs of fluorescent reporters facilitated the identification of clonal expansion of cells in a tissue is identified because the daughter cells of a particular clone combinatorically express the different pairs of fluorescent reporters of the parent cell. This powerful technology has been used to investigate neuronal network architecture [61,62,63], the expansion of crypt cells in the gastrointestinal tract [64], and endothelial cell proliferation [65,66,67]. A Brainbow 1.0 L transgenic strategy was used to identify that clonally dominant cardiomyocyte direct heart morphogenesis in Zebrafish hearts [68]. Sereti and colleagues created a *R26^VT2/GK^* transgenic mouse by a knock-in of a CAG promoter-driven Rainbow construct based on BrainBow principles into the Rosa26 locus [11]. Before Cre recombination, all cells express GFP. In the presence of Cre, the GFP is excised and Cerulean, mOrange, or mCherry fluorescent proteins are expressed stochastically. *αMHC-Cre::R26^VT2/GK^* drives the reporters in mouse cardiomyocytes from developmental stage E11.5 labeled neonatal cells with the predicted proportions of each reporter. *αMHC-CreER::R26^VT2/GK^* mice that drove expression of reporters in adult cardiomyocytes after Tamoxifen induction showed the postnatal cardiomyocytes had limited proliferative capacity in the absence of injury, consistent with prior investigations.

Of note, the efficiency of Cre-mediated recombination is a major consideration when using Rainbow/BrainBow/Confetti mice because the efficiency of recombination events can confound the quantification of proliferative events. For example, a small number of clonally expanded cells may be labeled and rare populations of cells may not be labeled using this strategy.

**Figure 7:**
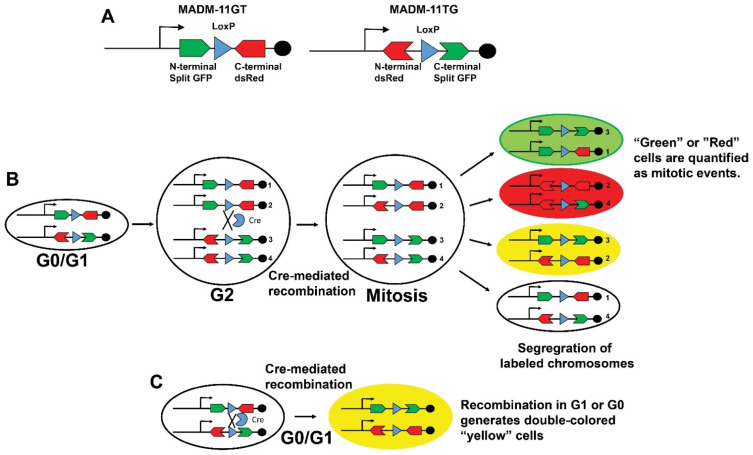
Mosaic Analysis with Double Markers (*MADM*). (**A**) Schematic of *MADM-11GT* and *MADM-11TG* transgenes that harbor spilt GFP and dsRed constructs with intronic LoxP sites. (**B**) Tamoxifen exposure induces the Cre recombinase expression that catalyzes an inter-chromosomal rearrangement of split fluorescent reporters and labels mitotic events. (**C**) Recombination during G1 or G1 produces double colored “Yellow” cells. The detection of “Green” or “Red” cells in panel (**B**) allows the quantification of mitotic events. Figure adapted from Zong, H. et al., Cell 2005, 121, 479–492.

Mosaic Analysis with Double Markers (*MADM*) mice. *MADM* mice are a powerful strategy to identify proliferation by marking cells that undergo mitosis [69,70]. The *MADM* mice harbor two split chimeric genes encoding the N-terminus of GFP and C-terminus of red fluorescent protein (dsRed) and N-terminus of dsRed and C-terminus of GFP. Each construct that has an intron containing a single LoxP site is targeted for homologous recombination to the identical loci of homologous chromosomes (Figure 7). There is no reporter expression in the absence of recombination. However, Cre-induced recombination between the LoxP sites restored GFP and dsRed expression in dividing cells after DNA replication at the G2 phase. Then, different outcomes are possible based on chromosome segregation into daughter cells during subsequent mitosis. The two recombinant sister chromatids can segregate into different daughter cells and express only GFP or dsRed, marking proliferated cells. Alternatively, the two recombinant sister chromatids segregate into one daughter cell with no fluorescence, similar to the parental cell. Finally, both recombinant chromatids segregate together to produce a daughter cell expressing GFP and dsRed. Of note, recombination in G1 or post-mitotic G0 generates cells that express both fluorescent reporters.

The *MADM* mice provide a readout of cytokinesis, a significant advantage when investigating cardiomyocytes because of endoreplication and multinucleation that occurs due to reentering the cell cycle. One limitation of the *MADM* strategy is the efficiency of labeling recombination events that can potentially underestimate the quantification of mitosis. Subsequent developments to improve the efficiency of labeling mitotic events included the creation of *MADM-ML* mice [71], which contains three mutually exclusive, self-recognizing LoxP variant sites as opposed to a single LoxP site present in the original *MADM* system. The initial *MADM* lines targeted the chimeric reports to chromosome 6 [70]. Recently, a genome-wide library of *MADM* mice has been created and validated to facilitate proliferation labeling [72].

The *MADM* technology has been used to quantify cardiomyocyte mitotic events [73,74,75]. Cardiomyocyte mitosis was readily observed during development, diminished in the first month of life, and was rare in uninjured adult *Myh6CreERT2(αMHC-Cre^ERT2^)::MADM-11^GT/TG^* mice [73]. Additionally, *Myh6CreERT2(αMHC-Cre^ERT2^)::MADM-11^GT/TG^* mice were used to confirm that pre-existing cardiomyocytes are the source of new cardiomyocytes after ischemic injury. More recently, *Myh6CreERT2(αMHC-Cre^ERT2^)::MADM-11^GT/TG^* mice have been used to examine the effects of Meis1–Hoxb13 double-knockout on cardiomyocyte mitosis [75], Pkm2 in cardiac regeneration [74], and Cdk1/CyclinB1 and Cdk4/CyclinD1 complexes (4F) to induce transient cardiomyocyte cycling [76].

Importantly, the *MADM* mouse technology has limitations in addition to the efficiency of labeling recombination events that can potentially underestimate the quantification of mitosis. The *MADM* strategy does not restrict Cre expression to only cells undergoing mitosis, limiting the ability to specifically ablate or express transgenes in specifically to these cells. Additionally, since *MADM* is based on the availability of a pair of *MADM* knock-ins between the gene of interest and the centromere, there is a need to generate knock-in cassettes for other chromosomes in order to investigate gene-specific function in the heart.

*αMHC-MerDreMer-Ki67p-RoxedCre* (*αDKRC*) mice. Advanced non-Cre DNA recombinases, such as Dre [77,78,79], VCre [80,81], SCre [80,81], and FLP [82,83] with DNA recognition sites other than LoxP, have unlocked the possibilities to engineer more sophisticated transgenic mice and interrogate temporal and lineage-specific cell biology [6,78,84]. Combining inducible non-Cre recombinases (MerDreMer) with modified Cre recombinases (RoxedCre) facilitates existing LoxP transgenics to answer previously unsolved questions in biology and disease.

**Figure 8:**
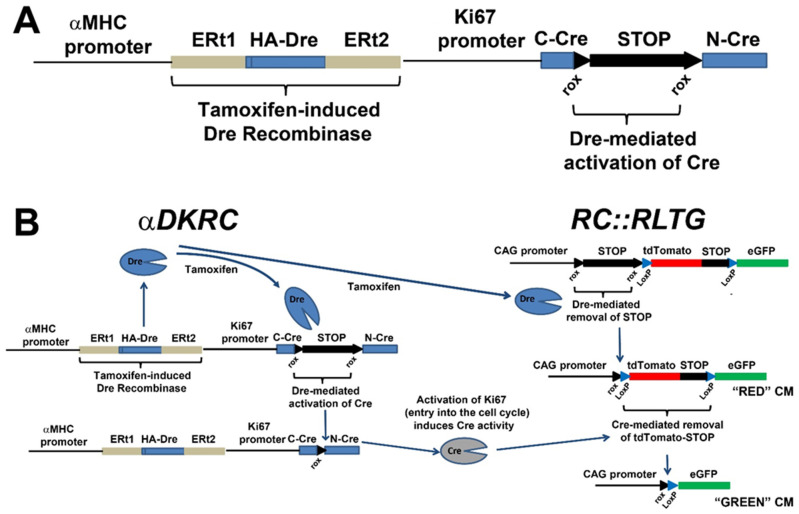
*αDKRC::RLTG.* (**A**) The *αDKRC* transgene. (**B**) Schematic outlining the expression of GFP in adult cycling cardiomyocytes. Tamoxifen exposure induces the cardiomyocyte-specific Dre-recombinase and subsequent excision of derived poly(A) signal repeats and flanked by two loxP sites from the RLTG reporter, resulting in the expression of tdTomato and RoxedCre to generate catalytically active Cre recombinase under control to the Ki67 cell cycle promoter. Cardiomyocytes reentering the cell cycle, as defined by activation of the Ki67 promoter, express Cre and excise the tdTomato-STOP cassette resulting in the expression of eGFP (enhanced green fluorescent proteins) in cardiomyocytes. A short duration of Tamoxifen exposure activates that reporter system. Schematic of expected changes in tdTomato and eGFP-labeled cardiomyocytes. Figure adapted from Bradley et al., Circ Res 2021, 128, 155–168.

Recently, we developed a new transgenic reporter mouse restricting Cre recombinase expression to adult cardiomyocytes that re-entered the cell cycle, as defined by the activation of a minimal Ki67 promoter [6] (Figure 8). Our goal was to create a transgenic mouse line that would facilitate the quantification and genetic manipulation of adult cycling cardiomyocytes and investigate these cells in the context of myocardial injury. To achieve this goal, we realized that tandem DNA recombinases recognizing distinct DNA motifs for recombination would be necessary to achieve the restricted expression of Cre. Additionally, the strategy needed to avoid the Rosa26 locus and be adaptable to existing LoxP transgenic mice to ensure the ability to use the vast resources of established transgenic mice for future investigations.

Therefore, we developed a transgenic approach based on the distinct specificities of Dre and Cre recombinases that recognized different DNA recombination sites sequentially. Dre recombinase is a Type I topoisomerase identified from a screen of P1-like bacteriophage and recognizes Rox DNA sites for recombination [77,79]. Dre recombinase is similar to Cre Recombinase; however, the Rox DNA sequences recognized by Dre are different from the LoxP DNA sites recognized by Cre recombinases, providing the ability to control recombination events. Dual Dre and Cre recombinases have been used to lineage trace cell fates, including the contributions of non-myocytes to myocytes [85], hepatocyte regeneration in liver injury [86], and neurogenesis [87]. Instead of using promoters to label cell lineages, we adapted the technology to create a new transgenic mouse αMHC promoter driving a Tamoxifen-inducible Dre recombinase in tandem with a minimal Ki67 promoter [88] that drives a Roxed-Cre, requiring Dre for the activation of Cre. The mouse harbors a *αMHC-MerDreMer-Ki67p-RoxedCre* transgene generated by conventional transgenesis, and nested, inverse PCR subsequently mapped the transgene to the endogenous αMHC region on chromosome 14, a genomic region that is transcriptionally active in cardiomyocytes. The *αDKRC* was then bred to *Rox-Lox-tdTomato-eGFP* (*RLTG*) mice that harbor tdTomato and eGFP promoters flanked by Rox and LoxP sites [89].

In adult *αDKRC::RLTG* mice, tamoxifen exposure induces the cardiomyocyte-specific Dre recombinase and subsequent excision of STOP cassettes of three identical SV40-derived poly(A) signal repeats and flanked by two loxP sites from the RLTG reporter, resulting in the expression of tdTomato and the generation of catalytically active Cre recombinase under control of the Ki67 cell cycle promoter. Then, any cardiomyocyte that reenters the cell cycle, as defined by activation of the Ki67 promoter, expresses Cre and excises the tdTomato-STOP cassette, resulting in the expression of eGFP (enhanced green fluorescent proteins).

Using *αDKRC* and Bromodeoxyuridine, we estimated ~0.07% and ~0.02% eGFP+ cycled cardiomyocytes per total cardiomyocytes per section after sixty minutes of LAD artery ligation-mediated ischemia and reperfusion myocardial infarction and sham groups, respectively [6]. The results were comparable to published estimates of cycling cardiomyocytes based on the incorporation of Bromodeoxyuridine or 5-Ethynyl-2-deoxyuridine and the expression of cell cycle markers [4,7,10,12,13,29,30,31,32]. Additionally, using *αDKRC/+::RLTG/RLTG* mice and examining cycling events across multiple short-axis sections of the myocardium suggests that endoreplication was the predominant outcome of cycling cardiomyocytes, with a ratio of 9:1 single to paired eGFP-positive cardiomyocytes.

The *αDKRC* mouse has a few important advantages. First, only a short duration of Tamoxifen exposure activates the reporter system, avoiding the potential problems of prolonged Tamoxifen that can confound experiments in terms of labeling and Tamoxifen-dependent effects of cardiac function [90]. For example, a ten-day course of Tamoxifen administration is sufficient to activate the *αDKRC* reporter system. However, some transgenic reporter systems, such as Brainbow/Confetti or *MADM*, require the presence of tamoxifen continuously to induce Cre activation during cycling that is needed to ensure appropriate labeling. Second, LoxP-based transgenic mice can be used with *αDKRC* to express transgenes or ablate endogenous genes, specifically in adult cardiomyocytes that reenter the cell cycle. The approach provides the ability to interrogate the contributions of specific signals to cardiomyocyte cycling after myocardial injury without affecting all cardiomyocytes. Third, *αDKRC::RLTG* mice offer the ability to potentially isolate cycled cardiomyocytes that are sparsely present for the characterization of differential gene expression. Fourth, *αDKRC::RLTG* facilitates the quantification of cycling cardiomyocytes, and proliferation or endoreplication can be inferred based on clustered or individual GFP-positive cardiomyocytes, respectively.

There are potential limitations of *αDKRC/+::RLTG/RLTG* mice. First, the activation of the 1.5 bp Ki67 promoter defines cycling. Although unlikely, a cardiomyocyte entering the cycle may fail to proceed through S-phase, leading to overestimating cycling events. Second, tamoxifen may not uniformly activate the *αDKRC* reporter across the entire myocardium, resulting in an underestimation of cycling cardiomyocytes. However, this is a limitation of all tamoxifen-dependent transgenic systems. Third, if Dre excised both LoxP and Rox-flanked cassettes, then a false-positive eGFP+ cardiomyocyte would be produced, potentially overestimating cardiomyocyte cycling. However, dual Dre and Cre approaches are rigorously specific enough for lineage-tracing transgenic models, as we validated in experiments in cell culture.

Recently, similar approaches using Dre and Cre DNA recombinases have been used to investigate the withdrawal cardiomyocyte cycling in preadolescent mice [91], supporting the power of tandem DNA recombinases to identify proliferating cells.

## 4. Future Directions

The evolution of transgenic technologies has improved the quantification and manipulation of cardiomyocyte cycling. Transgenic-based strategies have the potential to interrogate the unique molecular signatures of proliferating cardiomyocytes and their contributions to health and disease, with the promise of identifying mechanisms to promote regenerative medicine.

## References

[1] Benjamin E.J., Muntner P., Alonso A., Bittencourt M.S., Callaway C.W., Carson A.P., Chamberlain A.M., Chang A.R., Cheng S., Das S.R. (2019). Heart Disease and Stroke Statistics-2019 Update: A Report From the American Heart Association. Circulation.

[2] Yancy C.W., Jessup M., Bozkurt B., Butler J., Casey D.E., Drazner M.H., Fonarow G.C., Geraci S.A., Horwich T., Januzzi J.L. (2013). 2013 ACCF/AHA guideline for the management of heart failure: Executive summary: A report of the American College of Cardiology Foundation/American Heart Association Task Force on practice guidelines. Circulation.

[3] Eschenhagen T., Bolli R., Braun T., Field L.J., Fleischmann B.K., Frisen J., Giacca M., Hare J.M., Houser S., Lee R.T. (2017). Cardiomyocyte Regeneration: A Consensus Statement. Circulation.

[4] Alkass K., Panula J., Westman M., Wu T.D., Guerquin-Kern J.L., Bergmann O. (2015). No Evidence for Cardiomyocyte Number Expansion in Preadolescent Mice. Cell.

[5] Bergmann O., Zdunek S., Felker A., Salehpour M., Alkass K., Bernard S., Sjostrom S.L., Szewczykowska M., Jackowska T., Dos Remedios C. (2015). Dynamics of Cell Generation and Turnover in the Human Heart. Cell.

[6] Bradley L.A., Young A., Li H., Billcheck H.O., Wolf M.J. (2021). Loss of Endogenously Cycling Adult Cardiomyocytes Worsens Myocardial Function. Circ. Res..

[7] D’Uva G., Aharonov A., Lauriola M., Kain D., Yahalom-Ronen Y., Carvalho S., Weisinger K., Bassat E., Rajchman D., Yifa O. (2015). ERBB2 triggers mammalian heart regeneration by promoting cardiomyocyte dedifferentiation and proliferation. Nat. Cell Biol..

[8] Hesse M., Doengi M., Becker A., Kimura K., Voeltz N., Stein V., Fleischmann B.K. (2018). Midbody Positioning and Distance between Daughter Nuclei Enable Unequivocal Identification of Cardiomyocyte Cell Division in Mice. Circ. Res..

[9] Lin Z., von Gise A., Zhou P., Gu F., Ma Q., Jiang J., Yau A.L., Buck J.N., Gouin K.A., van Gorp P.R. (2014). Cardiac-specific YAP activation improves cardiac function and survival in an experimental murine MI model. Circ. Res..

[10] Senyo S.E., Steinhauser M.L., Pizzimenti C.L., Yang V.K., Cai L., Wang M., Wu T.D., Guerquin-Kern J.L., Lechene C.P., Lee R.T. (2013). Mammalian heart renewal by pre-existing cardiomyocytes. Nature.

[11] Sereti K.I., Nguyen N.B., Kamran P., Zhao P., Ranjbarvaziri S., Park S., Sabri S., Engel J.L., Sung K., Kulkarni R.P. (2018). Analysis of cardiomyocyte clonal expansion during mouse heart development and injury. Nat. Commun..

[12] Soonpaa M.H., Kim K.K., Pajak L., Franklin M., Field L.J. (1996). Cardiomyocyte DNA synthesis and binucleation during murine development. Am. J. Physiol..

[13] Soonpaa M.H., Koh G.Y., Pajak L., Jing S., Wang H., Franklin M.T., Kim K.K., Field L.J. (1997). Cyclin D1 overexpression promotes cardiomyocyte DNA synthesis and multinucleation in transgenic mice. J. Clin. Investig..

[14] Derks W., Bergmann O. (2020). Polyploidy in Cardiomyocytes: Roadblock to Heart Regeneration?. Circ. Res..

[15] Bergmann O., Bhardwaj R.D., Bernard S., Zdunek S., Barnabe-Heider F., Walsh S., Zupicich J., Alkass K., Buchholz B.A., Druid H. (2009). Evidence for cardiomyocyte renewal in humans. Science.

[16] Lazar E., Sadek H.A., Bergmann O. (2017). Cardiomyocyte renewal in the human heart: Insights from the fall-out. Eur. Heart J..

[17] Bergmann O., Zdunek S., Alkass K., Druid H., Bernard S., Frisen J. (2011). Identification of cardiomyocyte nuclei and assessment of ploidy for the analysis of cell turnover. Exp. Cell Res..

[18] Gerdes J., Lemke H., Baisch H., Wacker H.H., Schwab U., Stein H. (1984). Cell cycle analysis of a cell proliferation-associated human nuclear antigen defined by the monoclonal antibody Ki-67. J. Immunol..

[19] Gerdes J., Schwab U., Lemke H., Stein H. (1983). Production of a mouse monoclonal antibody reactive with a human nuclear antigen associated with cell proliferation. Int. J. Cancer.

[20] Scholzen T., Gerdes J. (2000). The Ki-67 protein: From the known and the unknown. J. Cell Physiol..

[21] Broad A.J., DeLuca J.G. (2020). The right place at the right time: Aurora B kinase localization to centromeres and kinetochores. Essays Biochem..

[22] Fu J., Bian M., Jiang Q., Zhang C. (2007). Roles of Aurora kinases in mitosis and tumorigenesis. Mol. Cancer Res..

[23] Willems E., Dedobbeleer M., Digregorio M., Lombard A., Lumapat P.N., Rogister B. (2018). The functional diversity of Aurora kinases: A comprehensive review. Cell Div..

[24] Hans F., Dimitrov S. (2001). Histone H3 phosphorylation and cell division. Oncogene.

[25] Wei Y., Yu L., Bowen J., Gorovsky M.A., Allis C.D. (1999). Phosphorylation of histone H3 is required for proper chromosome condensation and segregation. Cell.

[26] Carim S.C., Kechad A., Hickson G.R.X. (2020). Animal Cell Cytokinesis: The Rho-Dependent Actomyosin-Anilloseptin Contractile Ring as a Membrane Microdomain Gathering, Compressing, and Sorting Machine. Front. Cell Dev. Biol..

[27] Piekny A.J., Maddox A.S. (2010). The myriad roles of Anillin during cytokinesis. Semin. Cell Dev. Biol..

[28] Zhang L., Maddox A.S. (2010). Anillin. Curr. Biol..

[29] Heallen T., Morikawa Y., Leach J., Tao G., Willerson J.T., Johnson R.L., Martin J.F. (2013). Hippo signaling impedes adult heart regeneration. Development.

[30] Nakada Y., Canseco D.C., Thet S., Abdisalaam S., Asaithamby A., Santos C.X., Shah A.M., Zhang H., Faber J.E., Kinter M.T. (2017). Hypoxia induces heart regeneration in adult mice. Nature.

[31] Hirose K., Payumo A.Y., Cutie S., Hoang A., Zhang H., Guyot R., Lunn D., Bigley R.B., Yu H., Wang J. (2019). Evidence for hormonal control of heart regenerative capacity during endothermy acquisition. Science.

[32] Soonpaa M.H., Field L.J. (1997). Assessment of cardiomyocyte DNA synthesis in normal and injured adult mouse hearts. Am. J. Physiol..

[33] Katz E.B., Steinhelper M.E., Delcarpio J.B., Daud A.I., Claycomb W.C., Field L.J. (1992). Cardiomyocyte proliferation in mice expressing alpha-cardiac myosin heavy chain-SV40 T-antigen transgenes. Am. J. Physiol..

[34] Pasumarthi K.B., Nakajima H., Nakajima H.O., Soonpaa M.H., Field L.J. (2005). Targeted expression of cyclin D2 results in cardiomyocyte DNA synthesis and infarct regression in transgenic mice. Circ. Res..

[35] Soonpaa M.H., Zebrowski D.C., Platt C., Rosenzweig A., Engel F.B., Field L.J. (2015). Cardiomyocyte Cell-Cycle Activity during Preadolescence. Cell.

[36] Brodskiǐ V.I.A., Uryvaeva I.V., Brodskiǐ V.I.A. (1985). Genome Multiplication in Growth and Development: Biology of Polyploid and Polytene Cells.

[37] Ovrebo J.I., Edgar B.A. (2018). Polyploidy in tissue homeostasis and regeneration. Development.

[38] Fox D.T., Duronio R.J. (2013). Endoreplication and polyploidy: Insights into development and disease. Development.

[39] Lee H.O., Davidson J.M., Duronio R.J. (2009). Endoreplication: Polyploidy with purpose. Genes Dev..

[40] Orr-Weaver T.L. (2015). When bigger is better: The role of polyploidy in organogenesis. Trends Genet..

[41] Shu Z., Row S., Deng W.M. (2018). Endoreplication: The Good, the Bad, and the Ugly. Trends Cell Biol..

[42] Adler C.P., Friedburg H. (1986). Myocardial DNA content, ploidy level and cell number in geriatric hearts: Post-mortem examinations of human myocardium in old age. J. Mol. Cell Cardiol..

[43] Beltrami C.A., Di Loreto C., Finato N., Yan S.M. (1997). DNA Content in End-Stage Heart Failure. Adv. Clin. Path.

[44] Herget G.W., Neuburger M., Plagwitz R., Adler C.P. (1997). DNA content, ploidy level and number of nuclei in the human heart after myocardial infarction. Cardiovasc. Res..

[45] Cappella P., Gasparri F., Pulici M., Moll J. (2008). A novel method based on click chemistry, which overcomes limitations of cell cycle analysis by classical determination of BrdU incorporation, allowing multiplex antibody staining. Cytom. A.

[46] Leif R.C., Stein J.H., Zucker R.M. (2004). A short history of the initial application of anti-5-BrdU to the detection and measurement of S phase. Cytom. A.

[47] Benmaamar R., Pagano M. (2005). Involvement of the SCF complex in the control of Cdh1 degradation in S-phase. Cell Cycle.

[48] Nishitani H., Lygerou Z., Nishimoto T. (2004). Proteolysis of DNA replication licensing factor Cdt1 in S-phase is performed independently of geminin through its N-terminal region. J. Biol. Chem..

[49] Sakaue-Sawano A., Kurokawa H., Morimura T., Hanyu A., Hama H., Osawa H., Kashiwagi S., Fukami K., Miyata T., Miyoshi H. (2008). Visualizing spatiotemporal dynamics of multicellular cell-cycle progression. Cell.

[50] Vodermaier H.C. (2004). APC/C and SCF: Controlling each other and the cell cycle. Curr. Biol..

[51] Wei W., Ayad N.G., Wan Y., Zhang G.J., Kirschner M.W., Kaelin W.G. (2004). Degradation of the SCF component Skp2 in cell-cycle phase G1 by the anaphase-promoting complex. Nature.

[52] Zachariae W., Nasmyth K. (1999). Whose end is destruction: Cell division and the anaphase-promoting complex. Genes Dev..

[53] Niwa H., Yamamura K., Miyazaki J. (1991). Efficient selection for high-expression transfectants with a novel eukaryotic vector. Gene.

[54] Abe T., Sakaue-Sawano A., Kiyonari H., Shioi G., Inoue K., Horiuchi T., Nakao K., Miyawaki A., Aizawa S., Fujimori T. (2013). Visualization of cell cycle in mouse embryos with Fucci2 reporter directed by Rosa26 promoter. Development.

[55] Alvarez R., Wang B.J., Quijada P.J., Avitabile D., Ho T., Shaitrit M., Chavarria M., Firouzi F., Ebeid D., Monsanto M.M. (2019). Cardiomyocyte cell cycle dynamics and proliferation revealed through cardiac-specific transgenesis of fluorescent ubiquitinated cell cycle indicator (FUCCI). J. Mol. Cell Cardiol..

[56] Mort R.L., Ford M.J., Sakaue-Sawano A., Lindstrom N.O., Casadio A., Douglas A.T., Keighren M.A., Hohenstein P., Miyawaki A., Jackson I.J. (2014). Fucci2a: A bicistronic cell cycle reporter that allows Cre mediated tissue specific expression in mice. Cell Cycle.

[57] Basak O., van de Born M., Korving J., Beumer J., van der Elst S., van Es J.H., Clevers H. (2014). Mapping early fate determination in Lgr5+ crypt stem cells using a novel Ki67-RFP allele. EMBO J..

[58] Kretzschmar K., Post Y., Bannier-Helaouet M., Mattiotti A., Drost J., Basak O., Li V.S.W., van den Born M., Gunst Q.D., Versteeg D. (2018). Profiling proliferative cells and their progeny in damaged murine hearts. Proc. Natl. Acad. Sci. USA.

[59] Hesse M., Raulf A., Pilz G.A., Haberlandt C., Klein A.M., Jabs R., Zaehres H., Fugemann C.J., Zimmermann K., Trebicka J. (2012). Direct visualization of cell division using high-resolution imaging of M-phase of the cell cycle. Nat. Commun..

[60] Jang J., Engleka K.A., Liu F., Li L., Song G., Epstein J.A., Li D. (2020). An Engineered Mouse to Identify Proliferating Cells and Their Derivatives. Front. Cell Dev. Biol..

[61] Livet J., Weissman T.A., Kang H., Draft R.W., Lu J., Bennis R.A., Sanes J.R., Lichtman J.W. (2007). Transgenic strategies for combinatorial expression of fluorescent proteins in the nervous system. Nature.

[62] Cai D., Cohen K.B., Luo T., Lichtman J.W., Sanes J.R. (2013). Improved tools for the Brainbow toolbox. Nat. Methods.

[63] Lichtman J.W., Livet J., Sanes J.R. (2008). A technicolour approach to the connectome. Nat. Rev. Neurosci..

[64] Snippert H.J., van der Flier L.G., Sato T., van Es J.H., van den Born M., Kroon-Veenboer C., Barker N., Klein A.M., van Rheenen J., Simons B.D. (2010). Intestinal crypt homeostasis results from neutral competition between symmetrically dividing Lgr5 stem cells. Cell.

[65] Fioret B.A., Heimfeld J.D., Paik D.T., Hatzopoulos A.K. (2014). Endothelial cells contribute to generation of adult ventricular myocytes during cardiac homeostasis. Cell Rep..

[66] Gillich A., Zhang F., Farmer C.G., Travaglini K.J., Tan S.Y., Gu M., Zhou B., Feinstein J.A., Krasnow M.A., Metzger R.J. (2020). Capillary cell-type specialization in the alveolus. Nature.

[67] Manavski Y., Lucas T., Glaser S.F., Dorsheimer L., Gunther S., Braun T., Rieger M.A., Zeiher A.M., Boon R.A., Dimmeler S. (2018). Clonal Expansion of Endothelial Cells Contributes to Ischemia-Induced Neovascularization. Circ. Res..

[68] Gupta V., Poss K.D. (2012). Clonally dominant cardiomyocytes direct heart morphogenesis. Nature.

[69] Zong H. (2014). Generation and applications of MADM-based mouse genetic mosaic system. Methods Mol. Biol..

[70] Zong H., Espinosa J.S., Su H.H., Muzumdar M.D., Luo L. (2005). Mosaic analysis with double markers in mice. Cell.

[71] Henner A., Ventura P.B., Jiang Y., Zong H. (2013). MADM-ML, a mouse genetic mosaic system with increased clonal efficiency. PLoS ONE.

[72] Contreras X., Amberg N., Davaatseren A., Hansen A.H., Sonntag J., Andersen L., Bernthaler T., Streicher C., Heger A., Johnson R.L. (2021). A genome-wide library of MADM mice for single-cell genetic mosaic analysis. Cell Rep..

[73] Ali S.R., Hippenmeyer S., Saadat L.V., Luo L., Weissman I.L., Ardehali R. (2014). Existing cardiomyocytes generate cardiomyocytes at a low rate after birth in mice. Proc. Natl. Acad. Sci. USA.

[74] Magadum A., Singh N., Kurian A.A., Munir I., Mehmood T., Brown K., Sharkar M.T.K., Chepurko E., Sassi Y., Oh J.G. (2020). Pkm2 Regulates Cardiomyocyte Cell Cycle and Promotes Cardiac Regeneration. Circulation.

[75] Nguyen N.U.N., Canseco D.C., Xiao F., Nakada Y., Li S., Lam N.T., Muralidhar S.A., Savla J.J., Hill J.A., Le V. (2020). A calcineurin-Hoxb13 axis regulates growth mode of mammalian cardiomyocytes. Nature.

[76] Mohamed T.M.A., Ang Y.S., Radzinsky E., Zhou P., Huang Y., Elfenbein A., Foley A., Magnitsky S., Srivastava D. (2018). Regulation of Cell Cycle to Stimulate Adult Cardiomyocyte Proliferation and Cardiac Regeneration. Cell.

[77] Anastassiadis K., Fu J., Patsch C., Hu S., Weidlich S., Duerschke K., Buchholz F., Edenhofer F., Stewart A.F. (2009). Dre recombinase, like Cre, is a highly efficient site-specific recombinase in E. coli, mammalian cells and mice. Dis. Model. Mech..

[78] Han X., Zhang Z., He L., Zhu H., Li Y., Pu W., Han M., Zhao H., Liu K., Li Y. (2021). A suite of new Dre recombinase drivers markedly expands the ability to perform intersectional genetic targeting. Cell Stem Cell.

[79] Sauer B., McDermott J. (2004). DNA recombination with a heterospecific Cre homolog identified from comparison of the pac-c1 regions of P1-related phages. Nucleic Acids Res..

[80] Kishimoto K., Nakayama M., Kinoshita M. (2016). In vivo recombination efficiency of two site-specific recombination systems, VCre/VloxP and SCre/SloxP, in medaka (*Oryzias latipes*). Dev. Growth Differ..

[81] Suzuki E., Nakayama M. (2011). VCre/VloxP and SCre/SloxP: New site-specific recombination systems for genome engineering. Nucleic Acids Res..

[82] Gates C.A., Cox M.M. (1988). FLP recombinase is an enzyme. Proc. Natl. Acad. Sci. USA.

[83] Senecoff J.F., Bruckner R.C., Meyer-Leon L., Gates C.A., Wood E., Umlauf S.W., Attwood J.M., Cox M.M. (1986). Site-specific recombination promoted in vitro by the FLP protein of the yeast two-micron plasmid. Basic Life Sci..

[84] He L., Li Y., Li Y., Pu W., Huang X., Tian X., Wang Y., Zhang H., Liu Q., Zhang L. (2017). Enhancing the precision of genetic lineage tracing using dual recombinases. Nat. Med..

[85] He L., Huang X., Kanisicak O., Li Y., Wang Y., Li Y., Pu W., Liu Q., Zhang H., Tian X. (2017). Preexisting endothelial cells mediate cardiac neovascularization after injury. J. Clin. Investig..

[86] Wang Y., Huang X., He L., Pu W., Li Y., Liu Q., Li Y., Zhang L., Yu W., Zhao H. (2017). Genetic tracing of hepatocytes in liver homeostasis, injury, and regeneration. J. Biol. Chem..

[87] Sajgo S., Ghinia M.G., Shi M., Liu P., Dong L., Parmhans N., Popescu O., Badea T.C. (2014). Dre-Cre sequential recombination provides new tools for retinal ganglion cell labeling and manipulation in mice. PLoS ONE.

[88] Zambon A.C. (2010). Use of the Ki67 promoter to label cell cycle entry in living cells. Cytom. A.

[89] Plummer N.W., Evsyukova I.Y., Robertson S.D., de Marchena J., Tucker C.J., Jensen P. (2015). Expanding the power of recombinase-based labeling to uncover cellular diversity. Development.

[90] Bersell K., Choudhury S., Mollova M., Polizzotti B.D., Ganapathy B., Walsh S., Wadugu B., Arab S., Kuhn B. (2013). Moderate and high amounts of tamoxifen in alphaMHC-MerCreMer mice induce a DNA damage response, leading to heart failure and death. Dis. Model. Mech..

[91] Pu W., Zhang M., Liu X., He L., Li J., Han X., Lui K.O., He B., Zhou B. (2022). Genetic Proliferation Tracing Reveals a Rapid Cell Cycle Withdrawal in Preadolescent Cardiomyocytes. Circulation.

